# Atlantic salmon cardiac primary cultures: An in vitro model to study viral host pathogen interactions and pathogenesis

**DOI:** 10.1371/journal.pone.0181058

**Published:** 2017-07-20

**Authors:** Patricia A. Noguera, Bianka Grunow, Matthias Klinger, Katherine Lester, Bertrand Collet, Jorge del-Pozo

**Affiliations:** 1 Aquaculture and Marine Environment, Marine Scotland Science, Aberdeen, United Kingdom; 2 Fraunhofer Research Institution for Marine Biotechnology, Lübeck, Germany; 3 Institut für Anatomie-University Lübeck, Lübeck, Germany; 4 Royal Dick School of Veterinary Sciences - University of Edinburgh, Edinburgh, United Kingdom; Veterinary Pathology, SWITZERLAND

## Abstract

Development of Salmon Cardiac Primary Cultures (SCPCs) from Atlantic salmon pre-hatch embryos and their application as in vitro model for cardiotropic viral infection research are described. Producing SCPCs requires plating of trypsin dissociated embryos with subsequent targeted harvest from 24h up to 3 weeks, of relevant tissues after visual identification. SCPCs are then transferred individually to chambered wells for culture in isolation, with incubation at 15–22°. SCPCs production efficiency was not influenced by embryo’s origin (0.75/ farmed or wild embryo), but mildly influenced by embryonic developmental stage (0.3 decline between 380 and 445 accumulated thermal units), and strongly influenced by time of harvest post-plating (0.6 decline if harvested after 72 hours). Beating rate was not significantly influenced by temperature (15–22°) or age (2–4 weeks), but was significantly lower on SCPCs originated from farmed embryos with a disease resistant genotype (F = 5.3, p<0.05). Two distinct morphologies suggestive of an *ex vivo* embryonic heart and a *de novo* formation were observed sub-grossly, histologically, ultra-structurally and with confocal microscopy. Both types contained cells consistent with cardiomyocytes, endothelium, and fibroblasts. Ageing of SCPCs in culture was observed with increased auto fluorescence in live imaging, and as myelin figures and cellular degeneration ultra-structurally. The SCPCs model was challenged with cardiotropic viruses and both the viral load and the *mx* gene expression were measurable along time by qPCR. In summary, SCPCs represent a step forward in salmon cardiac disease research as an in vitro model that partially incorporates the functional complexity of the fish heart.

## Introduction

Farmed Atlantic salmon (*Salmo salar*) is one of the most important cold water diadromous species produced by aquaculture worldwide, exceeding 2.3 million tonnes in 2014 [1]. In the UK, Scotland’s record production in 2014 was associated with revenues of over £1.8 billion [2]. As with most species under intensive farming, disease management is one of the major challenges faced by the industry. Consequently, fish diseases and host-pathogen interaction remains among the top priorities for aquaculture research in Europe [3] and the UK [4].

Research in fin-fish infectious and emerging diseases has widely been performed in vivo, a costly approach requiring large numbers of fish and subject to variations between individuals [5]. Experimental work in aquaculture research involving live vertebrates has adopted the 3R principles (Replacement, Reduction and Refinement) as the framework for quality research. This is now incorporated into the European Directive 2010/63 and the amended UK Animals (Scientific Procedures) Act, 1986 (ASPA). It has also become a core requirement in the project authorisation process for fish experimental research, extended to the breeding, accommodation and care of fish as protected animals [6]. In this context, fish research is shifting the focus towards alternative approaches and procedures, such as *in vivo* non-lethal sampling [5,7] and in vitro models. All of these are capable of producing reliable results which can reduce the need for, or even replace, in vivo challenges.

Work on cell lines is a traditional in vitro approach, and lines derived from fish including salmonids, have been available and routinely used in diagnostics and research for many years [8]. Moreover, an in vitro model of rainbow trout (*Oncorhynchus mykiss*) satellite cells has recently been applied to study viral induced muscle atrophy due to *Salmonid alphavirus* (SAV) [9]. However, to understand some disease mechanisms, especially regarding host-pathogen interactions, cell monolayers are limited due to their lack of complexity [10–12].

Pathogenesis studies of Atlantic salmon disease potentially could be undertaken *in vivo* in other fish species; a commonly used approach in mammals. For example, the zebrafish (*Danio rerio*) has become a well-characterized and established vertebrate model [13–16]. Work on zebrafish has contributed enormously to the fields of genetics, developmental biology, toxicology, and biomedical research, specifically including cardiovascular studies [17–21]. The limitation of this approach for the study of salmonid disease is that zebrafish is a tropical fresh water species with optimum temperature at ~28°C, which makes it rather unsuitable for study of cold water piscine pathogens. This is also the case for other potential models such as medaka (*Oryzias latipes*) or fathead minnow (*Pimephales promelas*), among others.

Farmed Atlantic salmon is prone to numerous cardiotropic viral diseases, such as Cardiomyopathy Syndrome (CMS, induced by *Piscine myocarditis* virus), Heart and Skeletal Muscle Inflammation (HSMI, induced by *Piscine reovirus)*, Infectious Salmon Anaemia -ISA- (ISAV), and Pancreas Disease (PD, induced by *Salmonid alphavirus* also known as SPDV) [22–24]; the later also induces Sleeping disease (SD) in rainbow trout. Mortality due these conditions varies, but all of them can also cause severe growth rate reduction and downgrading at harvest, with consequential impact on commercial value. These infections exhibit differences in cardiac immunopathological responses [25,26] and some important insights into host pathogen interactions have been gained through in vitro studies on non-cardiac cell lines [12,27,28]. However, understanding the basis of cardiac diseases in fish, as with other hosts, has been hampered by the lack of appropriate models that mimic the complexity of the organ [29]. Paradoxically, despite extensive use of fish as models for applied research on non-piscine species, there is a relative lack of fish models for research on diseases of fish in commercial production.

Previously, self-contracting cardiomyocyte aggregates have been isolated from rainbow trout [30–32] and successfully used for human’s drug pharmacological testing [33]. We postulate that a similar model may be used for the study of salmon cardiotropic viruses. The primary aims of the present work were to establish, characterize and optimize the production of salmon cardiac primary cultures (SCPCs), using pre-hatch *S*. *salar* embryos and to evaluate their potential as an in vitro model for disease research, through challenges with fish viral agents of known cardiac tropism.

## Materials and methods

### Embryo sources and husbandry

Wild (W) and farmed (F) fertilized salmon eggs were sourced from a native Scottish river population (Don District Salmon Fishery Board hatchery), and from a breeding program routinely used by the aquaculture industry (AquaGen-Norway). Eggs were received at different maturation stages, recorded in accumulated thermal units (ATUs), a measurement system that incorporates the cumulative effect of temperature over time (each unit equals 1°C for 1 day). Batches of approximately 300 eggs were incubated at 5°C+/- 0.3°C in a 20L container with sterilised, aerated fresh water, replaced every second day.

### Egg processing and SCPCs isolation

Processing was initiated 24h from reception and continued until hatching, which takes place at approximately 480 ATUs. Briefly, eggs were removed from incubation in batches of 6 to minimise temperature-induced stress, and transferred, in water from the bath, to a laminar flow cabinet for processing. Whole embryos were swiftly dissected out of their chorion with sterilized scissors and tweezers. Embryos were immediately transferred to 1.5 ml Eppendorf tubes containing 0.5 ml Trypsin (2.5% Trypsin 10x Gibco by Life Technologies-NZ), were finely cut with scissors to facilitate digestion, and incubated for 1 minute. The embryos were further dissociated by vigorous pipetting. The tissue lysate was then transferred into 12 ml centrifuge tubes with 2 ml culture medium. This was composed of L15 medium (Lonza, UK) supplemented with 10% foetal bovine serum (Sigma-Aldrich, USA), 100 U/ML Penicillin and 0.1 mg/ml Streptomycin (Fisher Scientific, UK) (modified L15). The complete process lasted ~2 min.

The tubes were centrifuged for 5 minutes at 565g and 15°C, the culture medium removed and the pellet re-suspended in 2ml fresh modified L15 medium for plating. Material from each embryo was plated individually in x12 tissue culture plates (Cellstar-Greiner Bio-one-UK) and incubated at 22°C. Plates were examined daily for the following 3 weeks under a dissecting microscope (Motic-SMZ 168). Culture medium was renewed and non-attached debris was removed after 24h and weekly thereafter.

Developing SCPCs were recognized by their beating, and were harvested as soon as observed by dissecting them out using the tip of a 20μl pipette. They were individually transferred into x4 wells chambered glass slide or x8 chambered coverglass (Nunc^®^ Lab-Tek II, Thermo-Fisher-USA) for further culture. Modified L15 medium, refreshed weekly, was used for culture, and unless stated otherwise, SCPCs were cultured at room temperature (22°C). The culture time varied between experiments, as detailed below. The maximum survival time in culture was of 5 months, noted in SCPCs that were not used in any experiments.

### Histological and ultrastructural examination

For histological examination, SCPCs were fixed for 30 min with 10% formalin within the chambered slide. Addition of a drop of neutral red during fixation assisted with identifying the SCPCs during the process. Fixed SCPCs were collected and embedded in a pearl of HistoGel (Thermo-Scientific, USA), following the manufacturer’s instructions. Once the pellet was solidified, it was processed routinely within a biopsy bag inside a disposable CellSafe biopsy insert (Cellpath Ltd, UK). Processed material was embedded in wax, sectioned at 3μ and stained with Harris haematoxylin and eosin (H&E) or Ziehl Neelsen (ZN) by routine procedures. Slides were examined using an Olympus BX60 microscope.

For EM observation, SCPCs were fixed *in situ* by adding 0.5 ml cold Karnovsky’s fixative containing 2.5% glutaraldehyde and 2% paraformaldehyde in 0.1M phosphate buffer pH 7.4 (Electron Microscopy Sciences, USA) to the wells for 30 minutes at room temperature. Fixative was then replaced with 0.1M Phosphate buffer saline pH 7.4 (PBS), the sample transferred to a 1.5 ml Eppendorf tube and placed on a rocker for 15 min. This procedure was repeated twice followed by a further transfer to fresh buffer for short-term storage at 4°C. Post fixation was performed with 1% OsO_4_ in 0.1 M cacodylate buffer for 2 h at 4°C. Samples were then dehydrated through a graded series of ethanol and embedded in araldite resin (Fluka, Switzerland). Ultrathin sections were cut on an Ultracut E (LEICA, Germany) and stained with 0.5% uranyl acetate (Laurylab, France) and 3% lead citrate (Laurylab) in an Ultrastainer AC20 (LEICA, Austria). Grids were examined with a JEOL electron microscope JEM 1011 at 60 kV or JEM-1400 Plus at 120kV (JEOL, Japan).

### Fluorescent imaging

SCPCs were fixed *in situ* with 4% paraformaldehyde solution (ph7.4) for 15 min at room temperature, washed twice with (PBS) and then incubated with Alexa Fluor^®^ 488 Phalloidin (Life Technologies, USA) for filamentous actin (F-actin) according to manufacturer’s instructions. Chambers were dislocated from slides and coverslipped using Vectashield antifade mounting medium with DAPI for nuclear staining (VECTOR Laboratories Ltd. UK). Samples were visualised and imaged with a Zeiss confocal LSM700 or a Zeiss Axio Imager M2 upright microscope with fluorescence attachment.

### Viral infection and gene expression

Different virus isolates were used to test infectivity on the SCPCs model. One isolate of Infectious salmon anaemia virus (ISA 390–98 passage 8, 1.11 x10^8^ TCID_50_) and three isolates of *Salmonid alphavirus* subtype 1 (SAV-1) identified as F07-220 (passage 2, 3.5x10^5^TCID_50_, kindly donated by AFBI-Ireland), MS4640 (passage 7, 1.4x10^6^TCID_50_) and F93-125 (passage 13, 4.6x10^7^TCID_50_), representing agents with observed high, medium or no virulence on in vivo preliminary challenges. SCPCs were allowed to re-attach and acclimate for at least 48h before challenge. The final dose of infection was achieved by adding 1 or 0.5 ml of the virus aliquot directly into the chambered wells (100 or 50 ml respectively) to reach a hundred fold reduction of the inoculate concentration. To assess the effect of challenge dose, isolate F07-220 was also inoculated at a thousand fold reduction of the initial inoculate. After a 2 h absorption step, the culture medium was replaced in all chambers including the SCPCs exposed to culture medium only as negative controls. Single infected SCPCs or triplicates for each isolate were used in the evaluations of preliminary infection (7 days) and longitudinal infection (3 weeks), respectively. Incubation was performed at 15°C. At the end of the experiment, SCPCs were collected directly into 100μl lysis/binding buffer from the RNA extraction kit (see below), and immediately frozen at -80°C. For RNA extraction, samples were individually processed using a Dynabeads^®^ mRNA DIRECT^™^ Micro Kit (Catalog Number 61021, Ambion -Life technologies, USA) according to manufacturer’s instructions.

For quantification of *mx* and viral gene expression by qPCR, total RNA was first reverse transcribed into cDNA using the TaqMan Reverse Transcription Reagent kit (Applied Biosystems) as follows: 9.6 μl of total RNA and 1.25 μl 50 μM oligo-d(T)_16_ were mixed and heated to 70°C for 10 min and chilled on ice. The final volume was adjusted to 25 μl by adding Master mix comprised: 1x RT buffer (25 mM Tris–HCl pH 8.3, 37.5 mM KCl, 5.5 mM MgCl_2_), 0.5 mM each dNTP, 0.4 U RNase inhibitor and 1.25 U Multiscribe Reverse Transcriptase. The reaction was incubated at 48°C for 90 min, heat inactivated at 95°C for 5 min and stored at -80°C until use. Real-time PCR (RT-qPCR) assays were performed on a Roche LC480 System (Roche). TaqMan probes and primers to amplify the elongation factor 1α gene (elf), mx, SAV non-structural protein P1 (nsP1) and ISAV segment 8, are given in [12] and in [34]. One μl cDNA was added to the following mix contained in individual wells of a 96-well optical plate (Roche): 10 μl of TaqMan 2x PCR mix with UNG (Applied Biosystems), 8 μl of dH_2_O and 1 μl of a 20x mix containing forward primer (18 mM), reverse primer (18 mM) and probe (5 mM). The standard cycling conditions were 50°C for 2 min, 95°C for 10 min followed by 50 cycles of 95°C for 15 s and 60°C for 1 min. The crossing point (Cp) was determined by the maximum secondary derivative method and the values were converted into expression levels normalised against the reference gene, *elf*, using a standard curve. For preliminary infection, RT-qPCR was performed in triplicates on the same sample and the average result presented. For analysis of the kinetics of infection in longitudinal studies, the qPCR was performed on three infected SCPCs per time point (biological replicates). The sampling time points were 1, 7, 15, and 21 days post infection (dpi).

### Statistical analyses

The total efficiency of generation of SCPCs, expressed as the number of SCPCs isolated per embryo, was compared between different egg sources (wild *vs*. farmed). The SCPCs harvest efficiency variability due to embryological stage was assessed by comparing the numbers harvested at 380, 395, 405, 410, 415, 445 ATU, and the time of harvest post plating by comparison between 24-72h, 96-120h, >120h. Beating rates were expressed as the number of beats per minute. The effect of temperature and age on beating rate of cultured SCPCs was assessed by comparison between SCPCs kept at 15°C and 22°C, and between 2 week and 4 week old SCPCs. Beating rate differences were also assessed between two types of farmed eggs (IPN sensitive and resistant, Aquagen, UK). Most of the above described data were not normally distributed (Kolmogorov Smirnov test), and Kruskal-Wallis (KW) test was used for evaluation of the differences between the medians of each group, with a threshold of significance of p = 0.05. Exceptions to this were the evaluation of the effect of temperature on SCPCs beating rate, and simultaneous analysis of egg type and culture age, where the data were normally distributed. In this instance, a two sample t-test was used, with a threshold of significance of p = 0.05. A two-way ANOVA was used to analyse simultaneously the differences between IPN resistant vs. susceptible genotype egg source and culture age in a sample subset.

## Results

### Salmon cardiac primary cultures (SCPCs) isolation

Primary cultures of autonomously beating, three-dimensional heart tissue were successfully isolated from Atlantic salmon pre-hatch embryos and were maintained under laboratory conditions with minimal support up to 5 months post isolation. The method applied was repeatable and robust and several factors affecting the efficiency of this protocol were evaluated.

**Fig 1:**
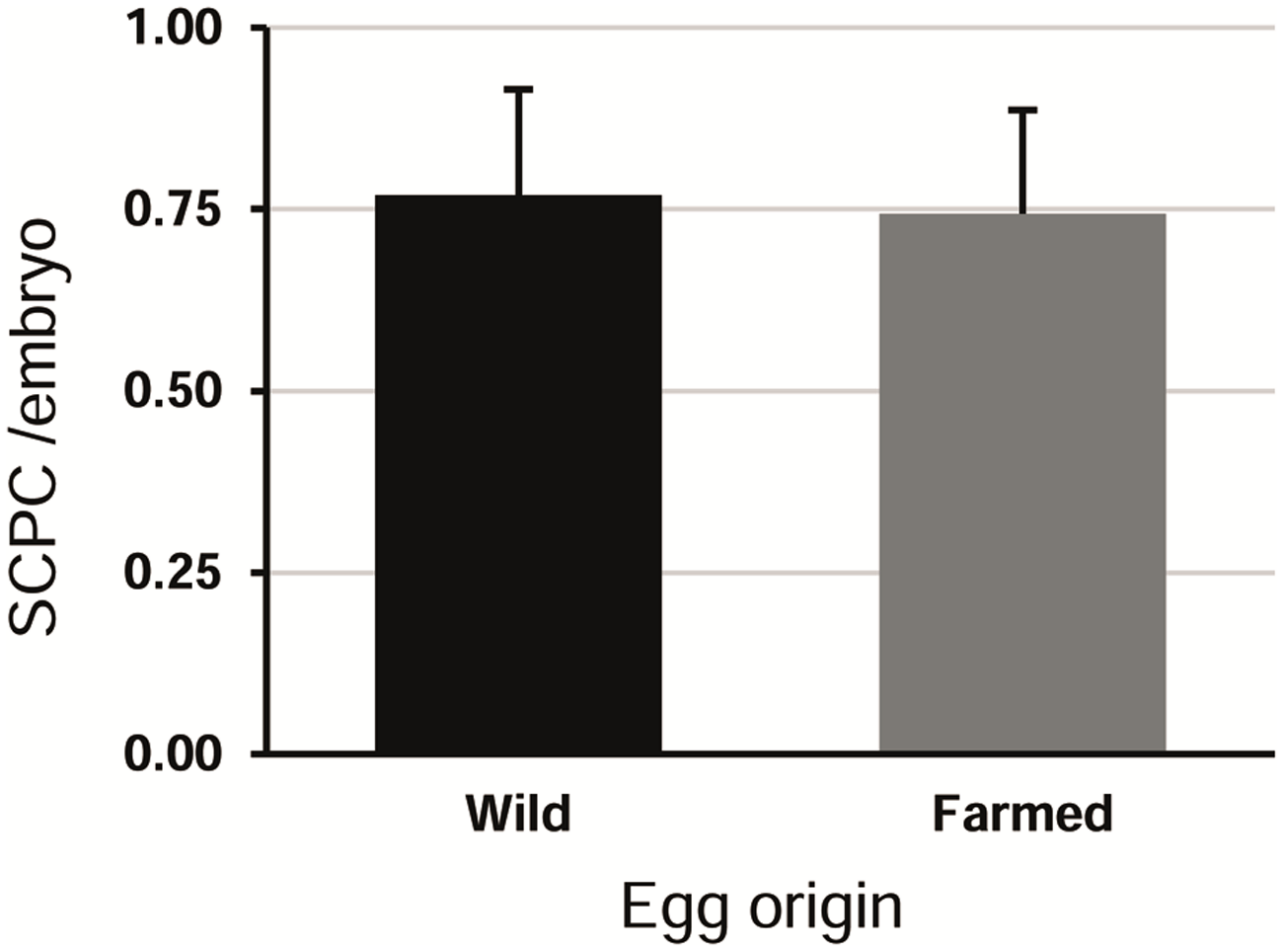
SCPCs generation efficiency by egg source. Generation efficiency was calculated as the number of SCPCs isolated per processed embryo. Wild n = 78, farmed n = 78; error bars = standard deviation; KW: H = 0.11, p = 0.74.

Eggs of farmed and wild origin resulted in similar total production efficiency (Fig 1) with no significant differences noted (KW, H = 0.11, p = 0.74).

**Fig 2:**
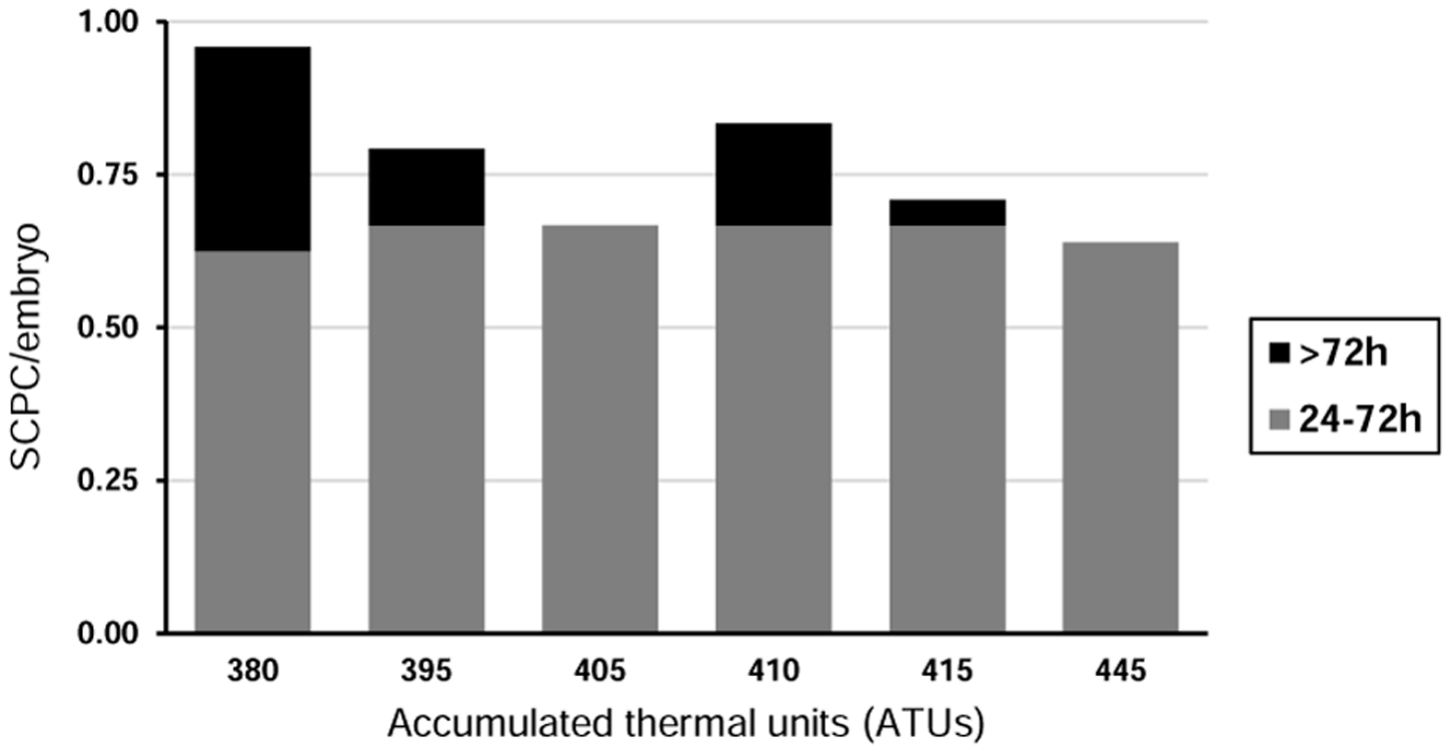
SCPCs generation efficiency by accumulated thermal units (ATUs) at processing and harvest timing (n_380-415ATU_ = 24; n_445ATU_ = 36). There is a small decrease in SCPC/embryo with increasing ATUs (descriptive only), while there is a large decrease when harvesting after 72h (KW: H = 8.64, p<0.003).

Embryonic developmental stage and timing of harvest after plating influenced SCPCs production efficiency for both farmed and wild eggs (Fig 2). SCPCs could be harvested from the isolation plate from 24h to 3 weeks from initiation of culture. Briefly, there was a small decline of up to 0.3 in efficiency with increasing ATU from ~380 to 445 ATUs (*i*.*e*. from reception and up to initiation of hatching), and a strong decline in efficiency (up to 0.6), when harvesting after 72 hours post-plating. It was noted that most SCPCs were visible and could be harvested before 36h.

**Fig 3:**
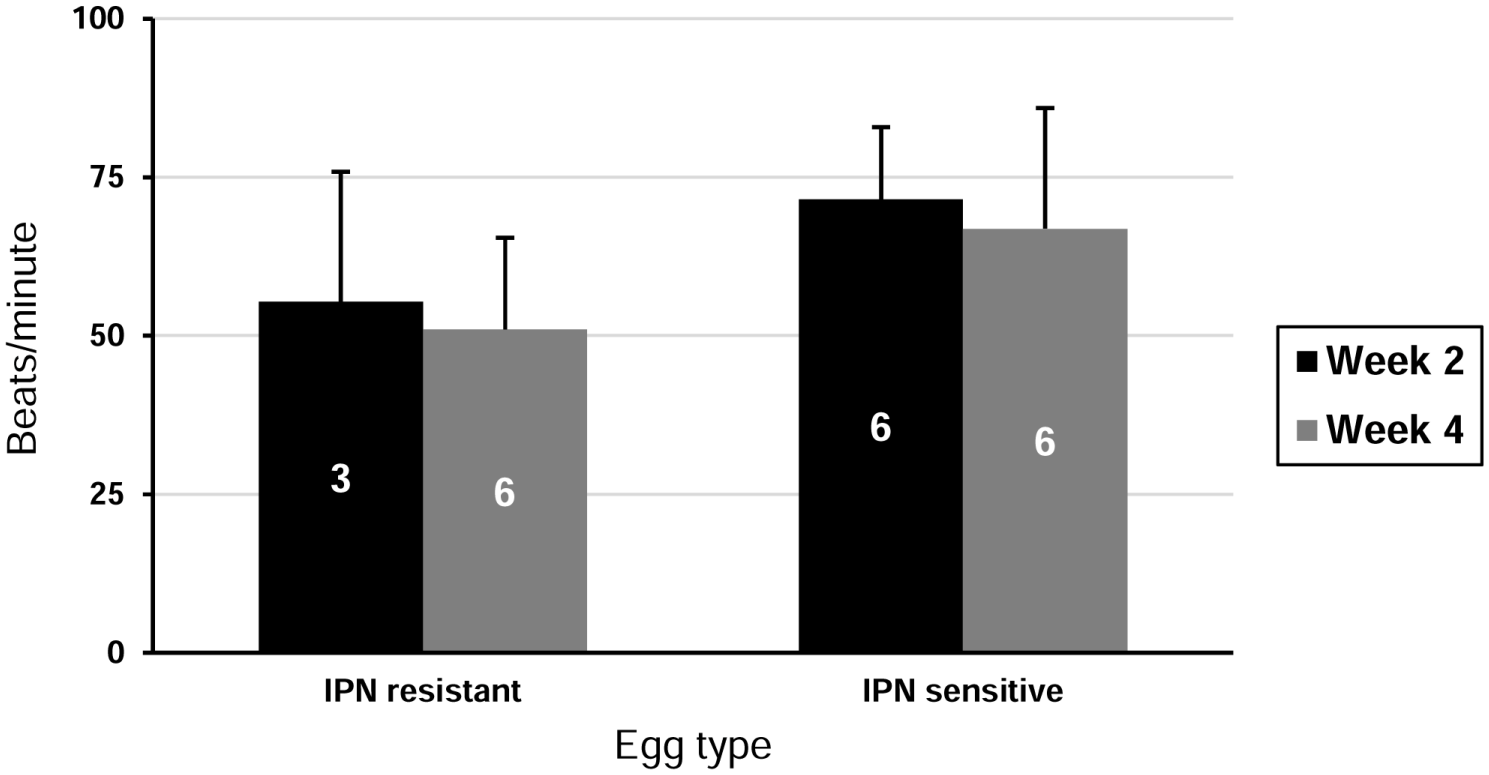
Effect of culture age and egg genotype on beating rate of SCPCs. Numbers within columns indicate total number of cultures used for each group. There is a significant difference in beating rate between IPN resistant and IPN sensitive eggs (two way ANOVA, F = 5.3, p<0.05), but not between week 2 and week 4 (two way ANOVA, F = 0.42, p = 0.53).

Several factors potentially influencing SCPCs beating rate were investigated. There was no significant difference in beating rate between SCPCs cultured at 15°C (51 ± 14.5 SD) and 22°C (50 ± 23 SD) (t-test: T = 0.12, p = 0.9), or between SCPCs at 2 weeks and 4 weeks post-isolation (KW, H = 0.03, p = 0.8). A two-way ANOVA was used to analyse simultaneously the differences between IPN resistant vs. IPN susceptible genotype egg source and culture age in a sample subset (Fig 3). Beating rate was significantly lower in SCPCs from eggs with an IPN resistant genotype (F = 5.3, p<0.05), with no differences due to culture age (F = 0.42, p = 0.53).

Several technical details were noted during the development of the protocol. The use of sterilized water for egg incubation helped to minimize fungal contamination during the incubation and, importantly, the subsequent plating of the embryonic tissues, thus reducing the need of antifungal agents in the culture medium. Occasionally it was noticed during isolation that if SCPCs–like cell aggregates were not beating, a gentle disturbance with a pipette tip or a needle could initiate beating activity. Post transfer survival was dependent on the capacity of the SCPCs for prompt re-attachment. Attachment was improved by forcing the SCPCs directly into the bottom of the well at transfer. This prevented it from being trapped at the surface of the culture medium by surface tension.

### Light and confocal microscopy

Two distinct SCPCs morphologies were recognised. The most common was suggestive of an *ex vivo* embryonic heart, while the less common was suggestive of *de novo* formation of discrete aggregates of self-contracting cells.

**Fig 4:**
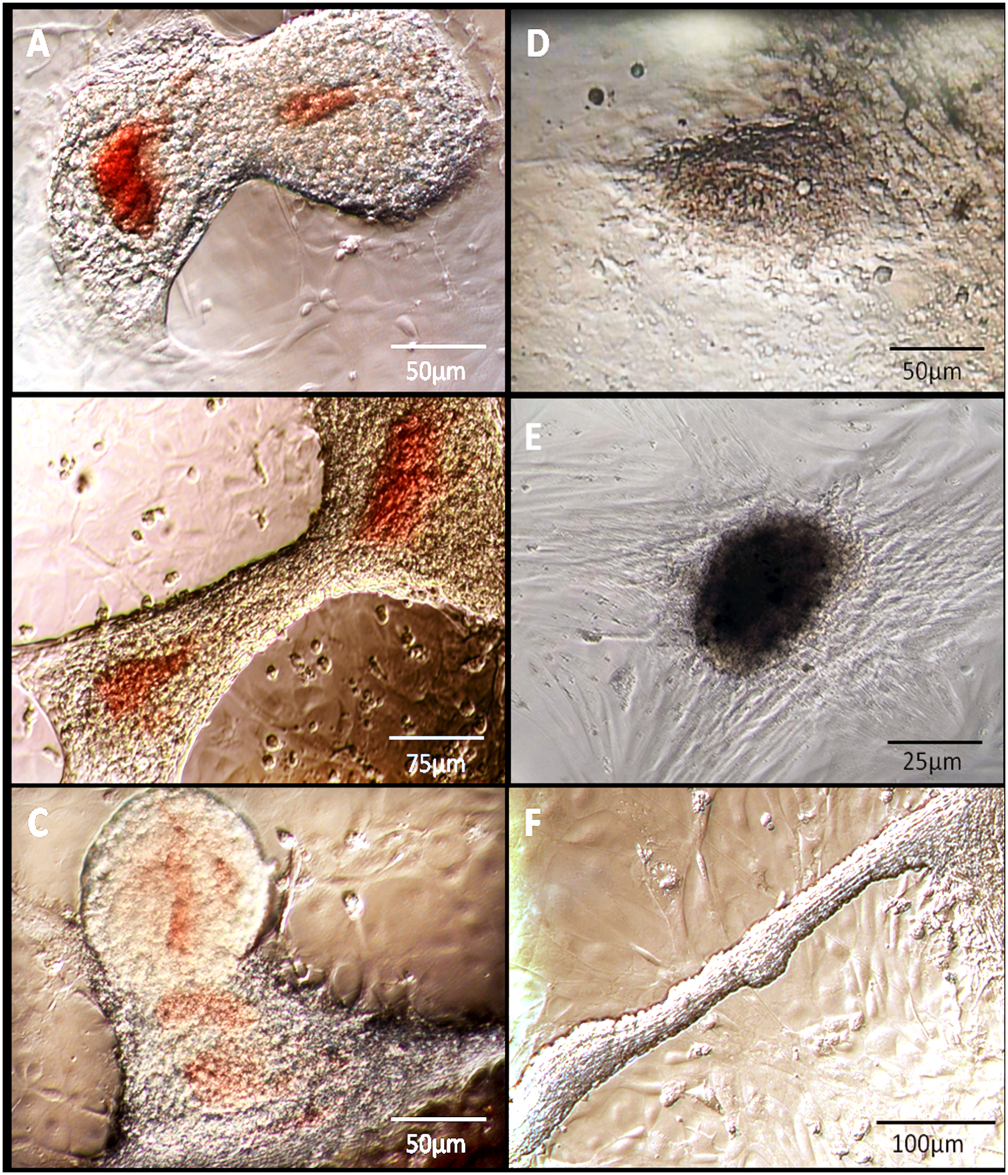
SCPCs morphological types as observed live under light microscopy: *Ex vivo* (A-C) and *de novo* (D-F). The *ex vivo* morphology features a characteristic double chambered structure containing blood cells (A), which may be retained during later culture stages (B-C). The *de novo* type is pleomorphic, commonly round to ovoid (D-E), and occasionally very elongated figures with a central non-attached section (F). Note a confluent surrounding cell monolayer is present in all types. Still images from video of unstained live SCPCs. Bright field microscopy with phase contrast.

Features of the embryonic heart, such as a double chambered structure filled with blood, are present in the *ex vivo* morphology, especially during the initial stages of culture. *Ex vivo* SCPCs are the first recognisable beating tissue after isolation (within the first hours), although the beating rate may take approximately 1 to 3 days to become established. After transfer to the culture plate from the isolation plate, *ex vivo* SCPCs develop a peripheral cell monolayer, with strong attachment to the well 1–2 days after transfer (Fig 4A–4C and S1 Video. Live SCPCs, ex vivo type).

The *de novo* morphology is pleomorphic and lacks blood-filled chambers. *De novo* SCPCs are most frequently flattened, beating, round to ovoid aggregates (Fig 4D and 4E and S2 Video. Live SCPCs, de novo type) but there are also less frequent, large elongated, solid, tubular forms (Fig 4F). The latter pattern features irregular attachment, and can display arrhythmic beating pace. Their occurrence is less frequent and characteristically harvested at later stages of incubation.

SCPCs size varies in relation to morphology and age. The *ex vivo* type is more consistent in size, measuring between ~300 to 500μm in the longest axis of the beating region at early development. The round to ovoid *de novo* SCPCs type is usually smaller at the same stage, while the elongated forms could be considerably larger (Fig 4F). After several weeks in culture, the *ex vivo* type can lose the initial morphological likeness to the embryonic heart (Fig 4B and 4C).

The attachment of SCPCs to the plate, dependent on the surrounding monolayer (composed of fibroblast-like and epithelioid cells), appears to be essential for the survival of the primary culture. All non-attached, floating SCPCs died within a week of culture.

**Fig 5:**
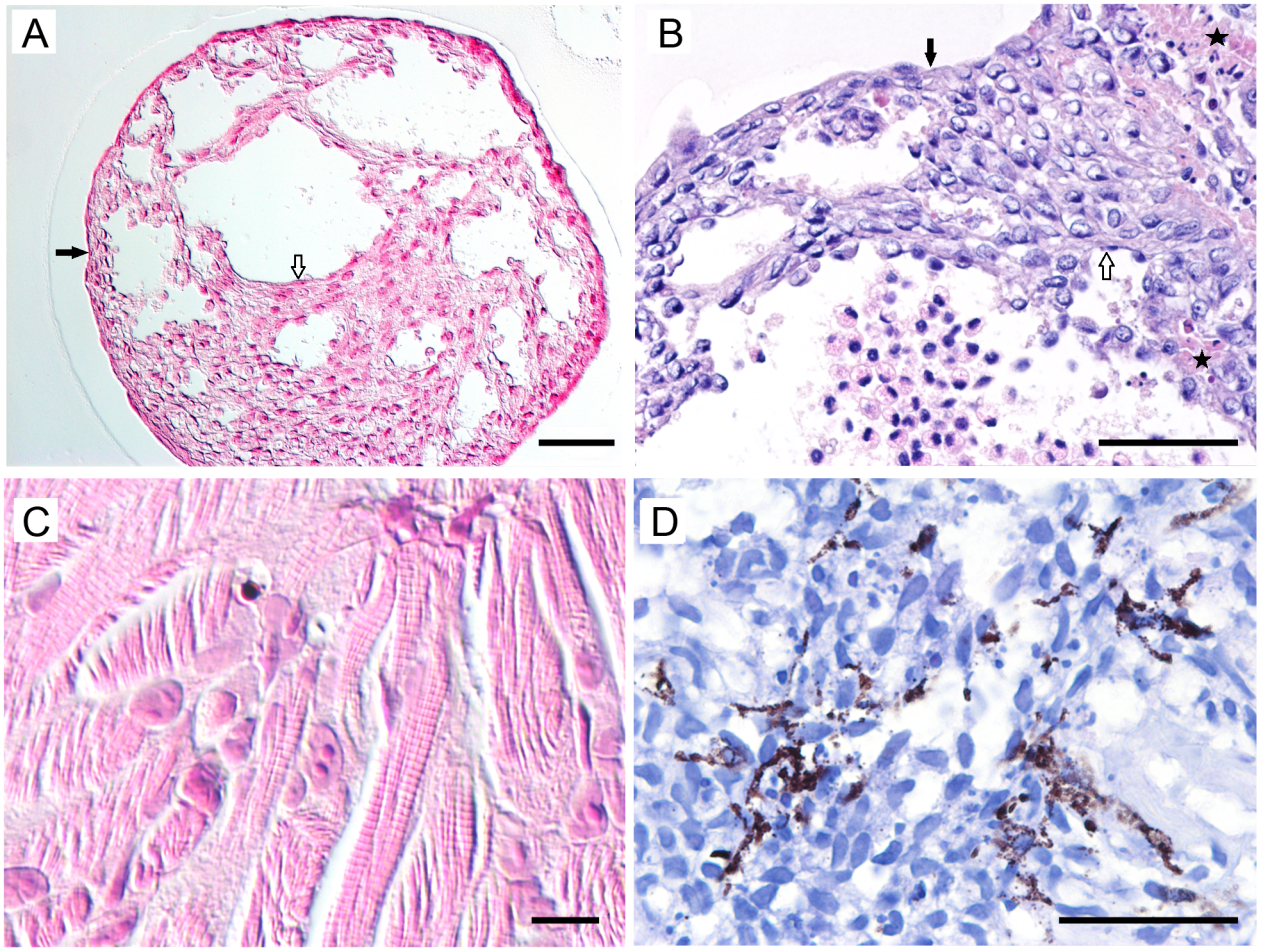
Histological sections of SCPCs. (A) Early stages of the *ex vivo* morphology shows a chambered structure with a trabecular arrangement of the myocardium, overlaid by a thin endocardial (white arrows)/epicardial layer (black arrows). (B) Note presence of pre-existing blood within chamber and areas of focal necrosis (stars). (C) In mature cultures of both *ex vivo* and *de novo* types the striation of the cardiac muscle becomes visible. (D) Non-acid fast, black, fine, extracytoplasmic and intracytoplasmic pigment granules are occasionally present (melanin). Haematoxylin and Eosin (A-C), Ziehl Neelsen (D). Scale bar = 50um (A, B, D), 10um (C).

Histologically (Fig 5), *ex vivo* SCPCs show a characteristic hollow structure with a lumen frequently -but not always- filled with erythrocytes and leukocytes (pre-existing blood). The wall is made of loose anastomosing trabeculae resembling spongy myocardium, which are mostly composed of spindloid cells with moderately defined cell borders, a central oval nucleus with watery to finely stippled chromatin, and a small amount of pale eosinophilic, wispy cytoplasm (myocardiocytes). Striation is not visible histologically in early cultures, but becomes visible in mature cultures (4-8weeks post isolation). Intercalating disks are not visible with light microscopy, but are visible ultrastructurally (image provided as S1 Fig. TEM of cardiomyocyte intercalated disc). The tissue resembling spongy myocardium is overlaid internally and externally by a thin layer of flattened cells (endocardium and epicardium, respectively),.. Histologically, *de novo* SCPC morphological type is structurally less defined, frequently forming solid, concentric arrangements where an inner region resembling a lumen is only rarely observed.

Focal cellular degeneration and necrosis were observed in some SCPCs in early young cultures. Similar degenerative changes, as well as accumulation of pale brown intra-cytoplasmic granular deposits, were observed in both early and “aging” SCPCs. With Ziehl Nielsen staining in an early culture, these granules were not acid fast (consistent with melanin granules).

**Fig 6:**
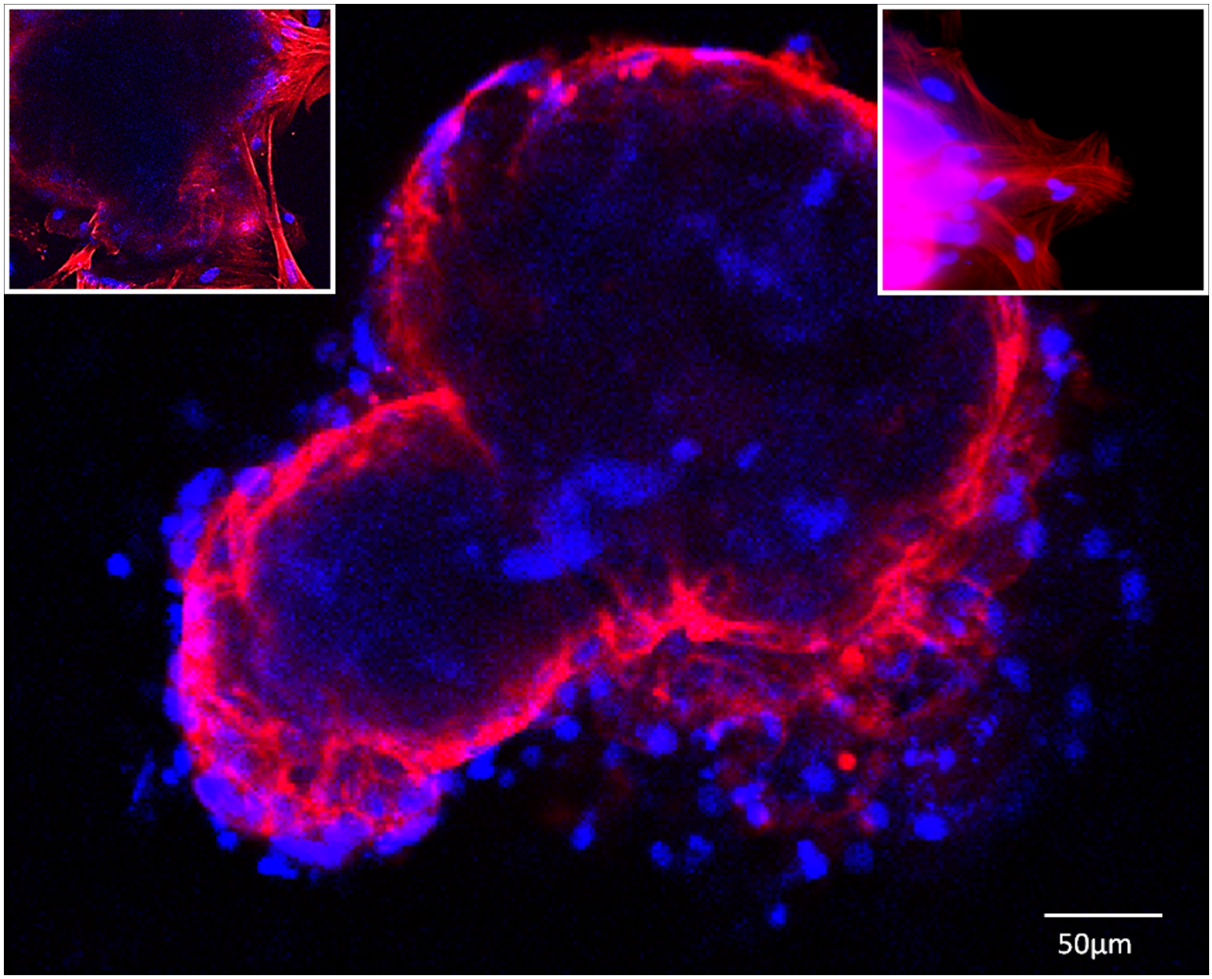
SCPCs *ex vivo* type under fluorescent confocal microscopy. The tridimensional morphology highlights a 2 chambered structure corresponding to the atrium and ventricle from the developing embryonic heart. Upper right and left corners inserts detail the edge zone where attachment occurs. Note elongated fibroblastic type cells anchoring the bulk of the SCPCs to the well bottom. Cytoskeleton F-actin filaments stained with Phalloidin (red) and nuclei with DAPI (blue).

*Ex vivo* SCPCs were examined with confocal microscopy (Fig 6), which revealed a bichambered tridimensional structure surrounded by a population of elongated, mesenchymal cells (possibly fibroblasts) at the attachment region and surrounding monolayer.

**Fig 7:**
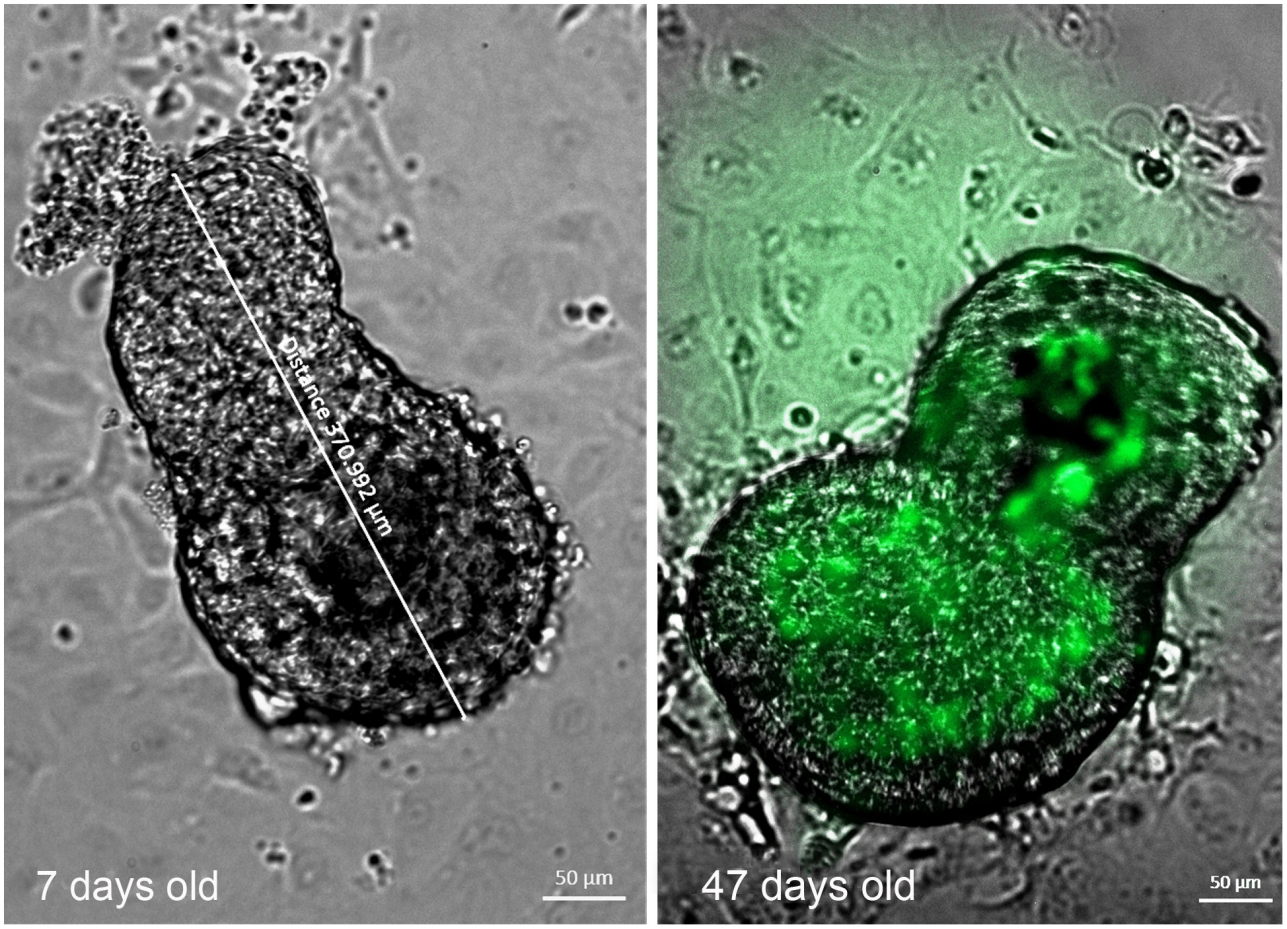
Live imaging of unstained SCPCs with fluorescent microscopy. Note increased auto fluorescence of SCPCs with increased culture age. Photographed with an Axio Imager M2, DIC superimposed fluorescence Green (Reflector 38HE Green fluorescent Protein with objective EC Plan-Neofluar 10x/ 0.30 M27).

There was evidence of cellular degeneration during culture. Non-stained mature cultures (>40 days old) showed increased autofluorescence suspected to be due to accumulation of lipofuscins during cellular aging (Fig 7).

### Electron microscopy

**Fig 8:**
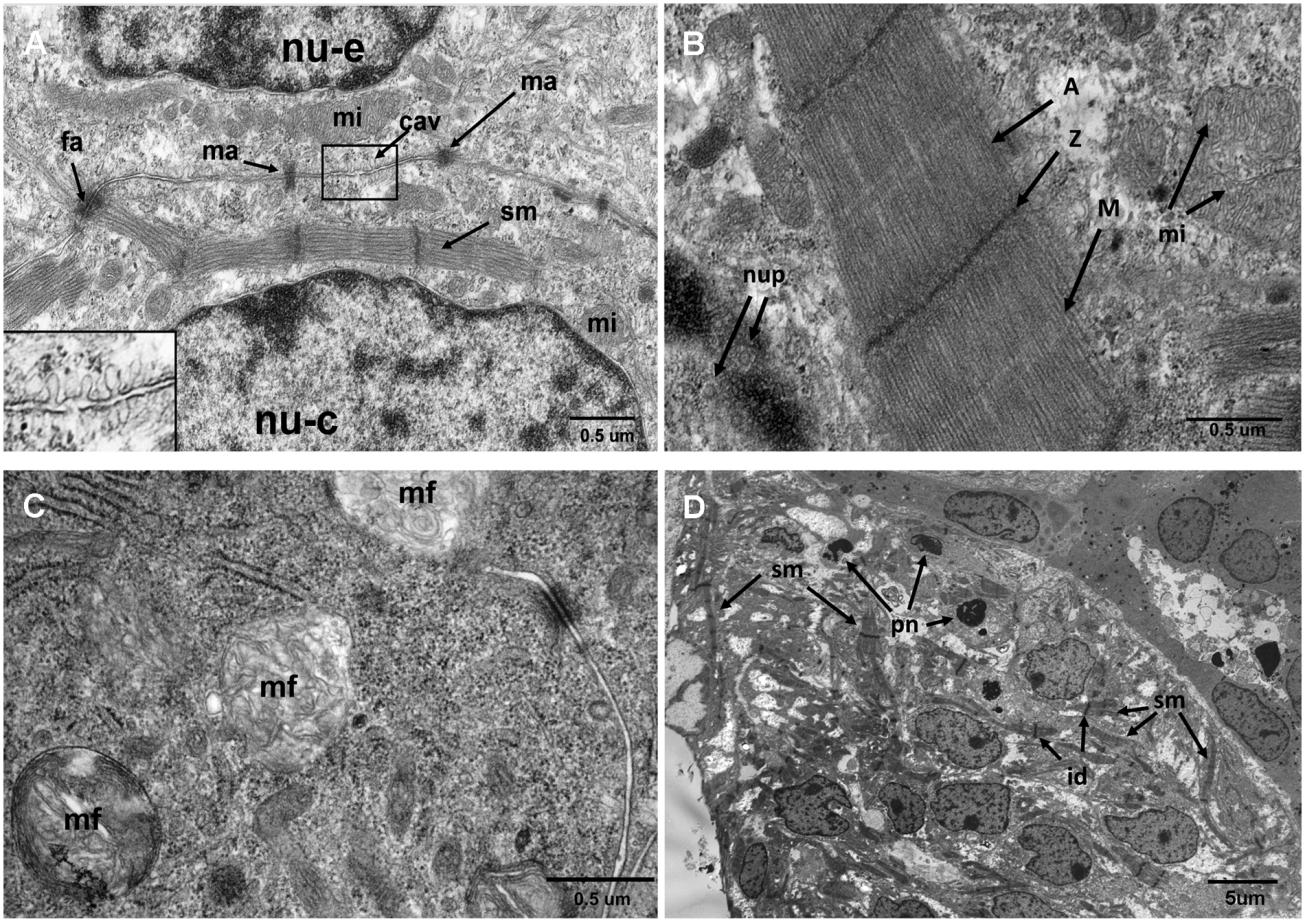
Ultrastructure of a beating 141 day old SCPCs. (A) an endothelia cell (top) overlays a myocyte (down);presence of fascia adherens (fa), desmosomes (macula adherens- ma) and cell membrane with caveolae (cav). Insert: detail of caveolae which are more numerous in the endothelia cell. (B) A sarcomere at maximum contraction shows the A-band, Z- and M-lines (A, Z, M), note also mitochondria (mi) and nucleus with pores (nup). (C) Abundance of intracytoplasmic myelin figures (mf). (D) There is evidence of focal degeneration, with widespread pyknotic nuclei (pn), sarcoplasmic rarefaction and fragmentation. Note that sarcomeres (sm) and intercalated discs (id) are retained (cardiomyocytes). Nuclei of endothelia cells (nu-e), nuclei of myocyte (nu-e), myofiber (mf), mitochondria (mi). TEM micrograph with a JEM 1011 at 60 kV and JEM-1400 Plus at 120kV.

Ultrastructural analysis performed in *ex vivo* SCPCs further confirmed the chambered structure with peripheral concentric cell layers and central lumen (image not shown). Both *ex vivo* and *de novo* morphologies feature cardiomyocytes with well-defined sarcomeres, abundance of mitochondria and tight junctions (macula adherens and fascia adherens; Fig 8B and 8C). There are cells ultrastructurally consistent with endothelium as suggested by the lack of intracytoplasmic sarcomeres and abundant presence of caveolae on the cytoplasmic membrane. At 141 days, features of stress, aging and degeneration were present, such as pyknotic nuclei, intracytoplasmic myelin figures (consistent with lipofuscin–Fig 8D).

### Preliminary infections

It was possible to infect SCPCs with a range of viral SAV isolates (both culture adapted and pathogenic during *in vivo* studies) as well as with one ISAV isolate.

**Fig 9:**
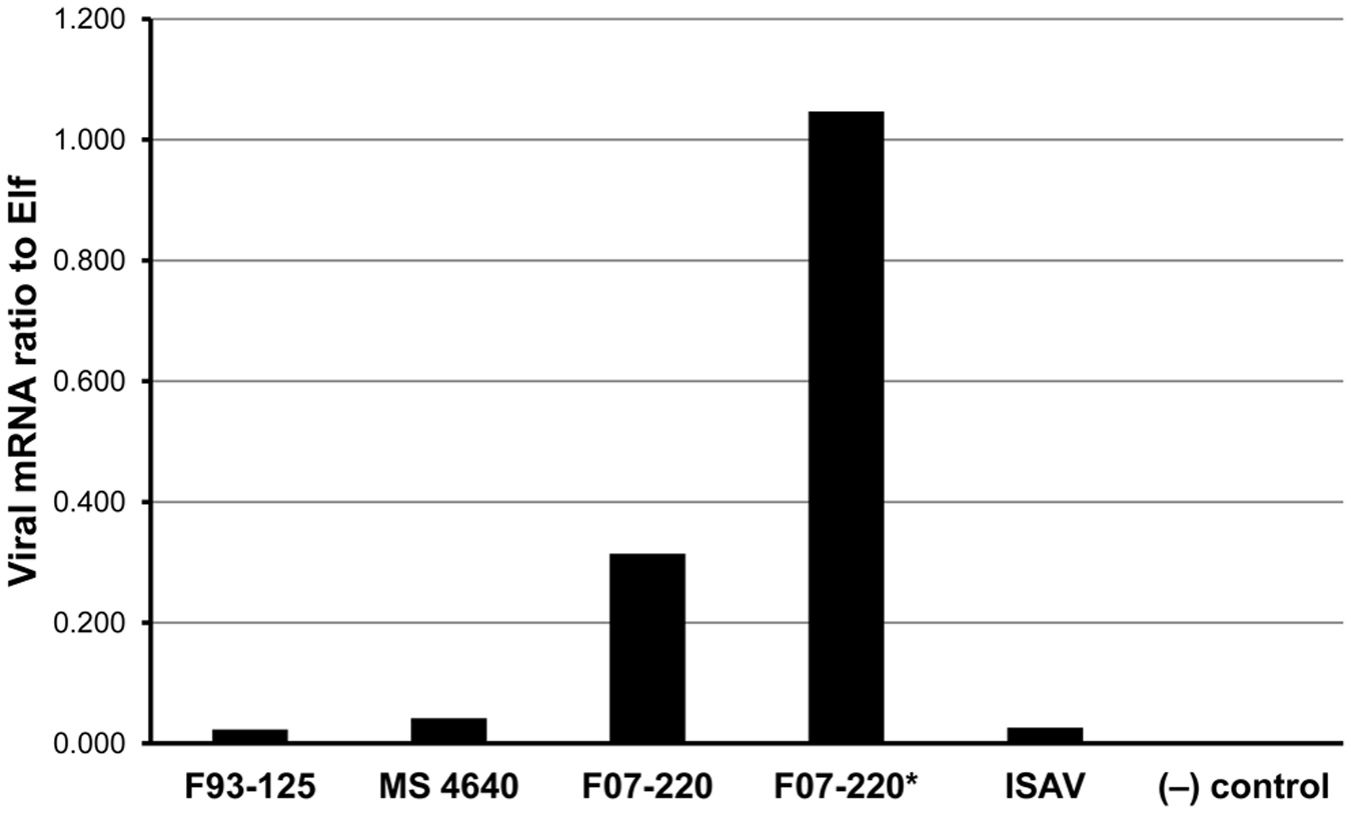
SCPC preliminary infection. Viral expression at 7 days post challenge with three different SAV and one ISAV isolate, expressed as the ratio of viral mRNA to e*lf*. Values are average of triplicate readings of single infected SCPCs.

The infection dose seems to have an effect on the amount of viral RNA detected, as observed in F07-220 challenge at two dilutions (Fig 9).

### Longitudinal infection studies

**Fig 10:**
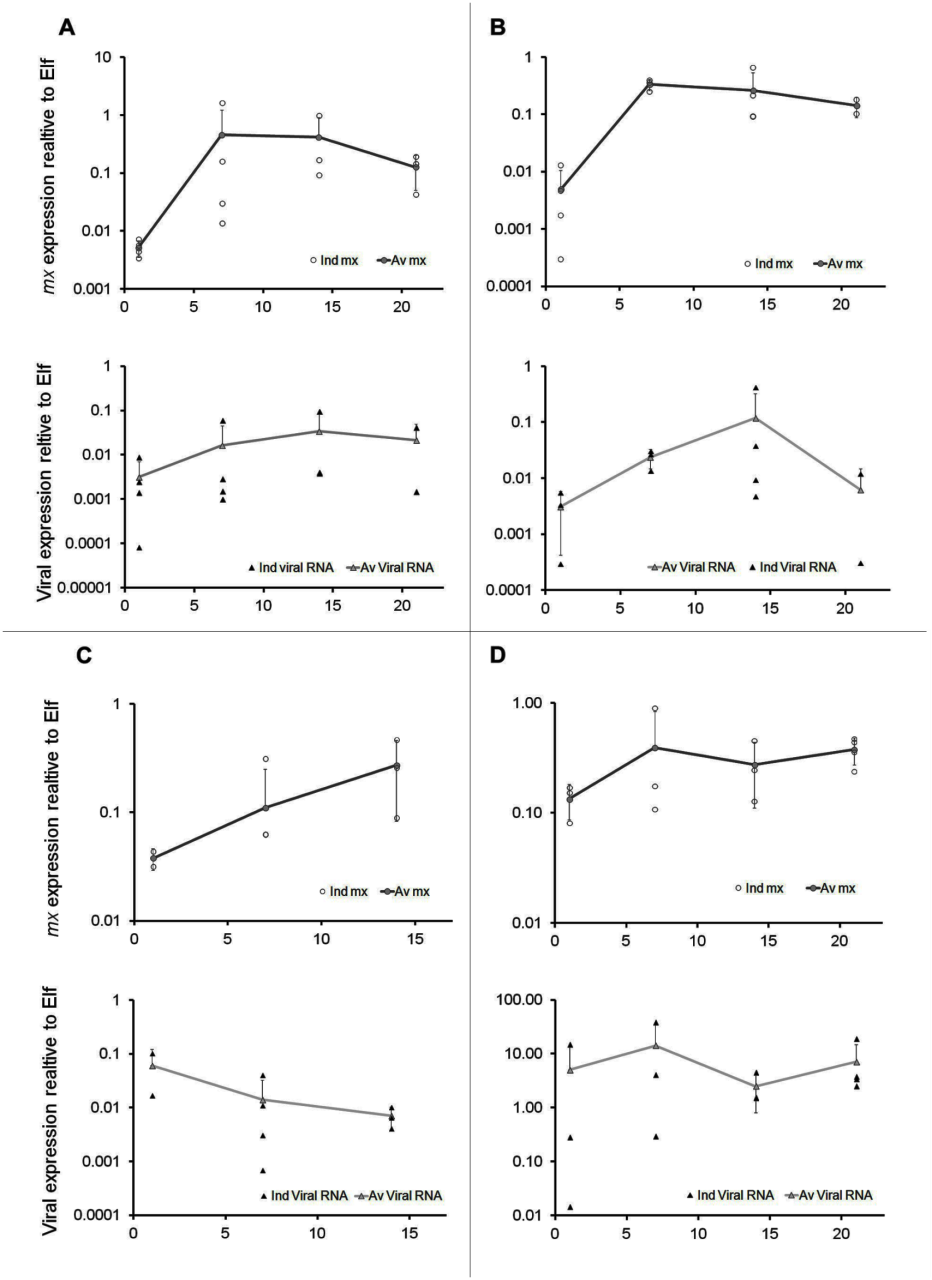
Kinetics of viral RNA and *mx* gene expression, for 4 different virus isolates in longer term challenge. A: F07-220 at 3.5x10^5^TCID_50_ (SAV), B: F93-125 at 4.6x10^7^TCID_50_ (SAV), C: MS4640 at 1.4x10^6^TCID_50_ (SAV), D: ISA 390–98 at 1.11 x10^8^ TCID_50_ (ISAV). Biological replicates (x3) average at each time point, individual data and SD, expressed as a ratio to the elongated factor (*elf*).

During longer term infections the variables associated with the kinetics of infection (viral RNA and mx gene expressions) showed to be measurable along time. A varying response in both variables was observed between isolates. The individual data at 0, 7, 14 and 21 days post infection for the viral protein nsP-1 and the expression of *mx*, relative to *elf* is shown in Fig 10. Data on isolate MS4640 covers 14 days post infection only as culture for day 21 was accidentally lost. Source data file is provided as supportive information (S1 QPCR data. Source for graphics of the kinetics of infection).

## Discussion

The reduced availability of wild-capture fish in the context of increased global demand for seafood, has resulted in continuous growth of the aquaculture industry worldwide [35]. In this context, the study of diseases affecting salmon, one of the most important species in cold water aquaculture, requires methodologies in agreement with bioethical principles seeking to investigate, facilitate and promote the advancement of alternative approaches [36]. The SCPCs model presented here is in line with the aim of this Directive to minimise the number of animals used in research while providing reliable results and allowing for optimal extrapolation into target species. Furthermore, non-hatched fish embryos are not classified as protected animals under UK regulations [37] and therefore, the approach poses no ethical conflict with regulations under the Animals (Scientific Procedures) Act 1986 (ASPA). On our work the SCPCs production efficiency per embryonated egg varied but on average, was not higher than 1. This compares favourably yet with egg to smolt survival of 70–80% under the greatly improved farmed conditions and only 0.15% to 3.2% among wild salmon [38–41]. Moreover, the cost of embryonated eggs compared to that of fry/parr or smolts, also contributes to a significant economical saving of the SCPCs approach.

Model organisms and in vitro approaches have made an enormous contribution to the basic understanding of the fundamental properties of all living cells [42] and currently, in vitro tissue and biological models based on fish are a well-established practice in human and veterinary medicine [43].

Since the early development of fish cell lines, fish research has benefited from adopting and adapting human and veterinary medicine approaches [44] and similar benefits can be expected for studies on disease mechanisms and host-pathogen interactions. As acknowledged for human medicine studies however, the genetic, molecular, cellular and immunological differences between target organisms and models can limit the effectiveness [45]. To closer replicate the events and processes taking place during infection and disease in a targeted host and organ, closer taxonomic proximity and higher levels of biological complexity can only be beneficial, i.e. using species -specific fish models.

In this scenario the establishment of fish as a widely used vertebrate model for human biomedical research, including cardiovascular studies [15,19], only occurred as the result of the cumulative evidence that, despite anatomical differences, there was a high degree of conservation of the molecular and cellular basis of relevant processes and mechanisms. In particular, fish heart models have been found to have a higher similarity to the characteristics of the human heart than the traditional murine models and have been therefore suggested as a functional substitute for in vitro toxicological testing [15,19–21,32].

In order to establish if the SCPCs approach was a suitable infection and disease model, it was necessary to streamline SCPCs production and establish a methodology for viral infection. Previous work with rainbow trout [30–32] showed the potential of the in vitro heart culture approach. It is generally recognised however that trout is a hardier species than salmon; the wider temperature tolerance of trout might contribute to its higher robustness [46].

Building on previous, successful work with rainbow trout, our results showed that Atlantic salmon embryos of wild and farmed origin could also be successfully used to generate tridimensional primary cultures of spontaneously beating heart tissues. SCPCs can be used as a species-specific model for host–pathogen interaction studies of Atlantic salmon cardiotropic agents.

The analysis of production efficiency indicators such as embryo development and SCPCs harvest time resulted in information to optimize the production of SCPCs. By focusing efforts in a specific embryo developmental period (initial 72h), it is possible to generate the numbers of SCPCs required for experimental work while reducing processing time and, importantly, produced homogenous and synchronously developing batches of SCPCs.

ATU values is a commonly used metric to track progress of incubating eggs and estimate the window of different developmental landmarks, such as “eyed egg” (embryo eye spots become visible), hatching point or embryo emergence [39]. ATU values at particular developmental stages are dependent upon temperature, differs between fish species and are further influenced by environmental factors [41]. Consequently, there can be variation between batches in the exact number of ATUs required to reach different landmarks. Therefore, the most efficient SCPCs production window (expressed in ATUs) for each batch of eggs will depend upon embryo maturity at the initiation of laboratory work, and the incubation conditions provided. In our work, we detected that eggs incubated at 5°C, demonstrated a slight steady decline in production efficiency with increasing embryo maturation between 380 and 445 ATUs, i.e. the last 2 weeks prior to hatching. This decline has minimal impact in the efficiency of SCPCs production while the timing of harvest post plating was a more important factor, with a significantly improved efficiency during the first 24-72h from plating (Fig 2). Although SCPCs could develop and be harvested up to 3 weeks from plating, we recommend focussing on the early window detailed above in order to obtain synchronously developing SCPCs of similar age.

Furthermore, the more structurally predictable *ex vivo* morphological type is the most common form in this initial period, further contributing to the general homogeneity even when they continue further development in vitro.

It is important to note that awareness of the *de novo* type being more frequently encountered during later stages of incubation, although not used for this particular study, could contribute to experimental planning as they may be suitable for other type of studies.

SCPC beating rate may be useful as a proxy for cellular viability or degeneration during challenge studies. In our work, the beating rate varied between individual SCPCs, but, overall, there was only a significant effect of egg source (ANOVA, F = 5.3, p < 0.05), while age (weeks) did not have a significant effect (ANOVA, F = 0.42, p = 0.53) on beating rate. The SCPCs obtained from eggs without a QTL for resistance to IPN disease (labelled S = sensible to disease), beat at a higher rate, irrespective of the age of the culture. This suggests that the batch/egg source among farmed origin ones, significantly influences the variability of beating rate which should be taking into account in the design of studies planning to use this variable.

The ability to maintain SCPCs under laboratory conditions for several months with minimal support is a key feature of the model considering the flexibility it provides to experimental timetables. Similar longevity had been reported for rainbow trout self-contracting cardiomyocyte cultures [30,31,33], evaluated on the retention of the beating capacity. The persistence of beating capacity in aging SCPCs suggests that “beating” is a resilient feature that can be relied upon during long term studies. For example, age-dependent susceptibility is a well-known characteristic in mammalian Alpha virus infections whereby more severe disease is observed in younger hosts [47,48], and this may be replicated by the model. Similarly, in Sindbis virus (SINV) infections in mice, the mechanism of age-dependent virulence is due to the induction of neuronal apoptosis [49] and this feature has been found to be reflected also in cell culture infections [50]. The SCPCs culture ageing could therefore represent a positive feature, widening its applications into testing potential age-dependent susceptibility to infection.

SCPC ageing occurs during culture. The results of the present study with SCPCs derived from *S*. *salar*, shows that a process of degeneration develops during culture, despite the fact that beating capacity is retained for several months. This is supported by increased auto-fluorescence (AF) of live SCPCs recorded during culture (Fig 7) and ultra-structural observations on 141 days old SCPCs showing increased myelin figures and other cell degenerative changes (Fig 8). These findings are consistent with lipofuscin accumulation within SCPCs.

Lipofuscins are the most commonly described AF compounds, frequently referred as “age pigments” [51]. They are due to the accumulation in endo-cytoplasmic granules of undigested cell materials resulting from phagocytosis and autophagy processes [52]. Several mechanisms apart from ageing can be involved in lipofuscin accumulation, such as disruptions in autophagy, lysosomal alteration or oxidative damage [53]. Other storage bodies referred to as ceroid pigments have similar AF properties and accumulate as a result of specific pathological conditions or experimental manipulations [51].

AF is frequently seen as a complication, in experiments especially when labelling with exogenous fluorochromes are required (lipofuscin is auto auto-fluorescent). However, AF also represents a useful biological tool as the endogenous fluorophores act as intrinsic biomarkers, strictly influenced by the morphological and metabolic conditions of the cells [54]. Therefore, the AF signal can be used for monitoring altered physiological or morpho-functional properties. Overall, the influence of SCPCs degenerative changes during culture and the resilience of their beating capacity, require further investigation to explore their potential usefulness, for example, its application in long term longitudinal studies. Occasionally, black, intracytoplasmic and extracytoplasmic pigment granules were noted histologically. These granules were not acid fast and are consistent with melanin granules.

SCPCs can be used in controlled viral challenges. The model was tested with different cardiotropic viral agents resulting in infection and replication. In spite of the small size of the SCPCs, the immune gene expression (*mx* gene) of infected SCPCs and the virus marker could be measured simultaneously by molecular approaches (Fig 9). Challenge using different virus types and isolates induced different responses, with all triggering an immediate increase of the expression of the *mx gen*, resulting in different outcomes in the viral expression response. Among the SAV isolates, only MS4640, showed a constant decreasing trend in the viral protein expression. over a 2 weeks period In contrast, F93-125 (a culture adapted isolate), demonstrated an increase before returning to initial levels by week 3 post challenge and F07-220 (a highly virulent isolate *in vivo*), showed a steady increasing trend in spite of the initial *mx* gene induction, which decreased after a week.

ISAV is a notifiable agent for the World Organization of Animal Health (OIE) and represent a very different virus type. SCPCs infection with ISAV led to an increase in the viral RNA expression during the first week, followed by a lower but maintained level of expression for up to 3 weeks in the presence of a stable *mx* gene induction.

When exploring pathogenesis, virus tropism is a very important factor and currently available fish cell lines are not permissive to infection with all ISAV strains. In vitro replication has been demonstrated in only a few cell lines (derived mainly from Atlantic salmon), such as salmon head kidney (SHK-1), Atlantic salmon kidney (ASK), Atlantic salmon (AS) and in head kidney leukocytes (TO) [55], while other routinely used cell lines tested appearing refractory to ISAV [56]. ISAV induces circulatory disturbances and has been shown to infect gill epithelial cells [55] and have a non cytolytic endothelio-tropism [57]. The SCPCs model was sensitive to ISAV (ISA 390–98 isolate) infection and the presence of endothelial tissue further suggest that the model could be used for the studies on ISAV pathogenesis. Visualization of this virus in the endothelia by immunofluorescence constitutes an area for future research.

The histopathological changes associated with infection by *Salmonid alphavirus* have been well described. Specifically, the heart develops focal to diffuse cardiomyocyte necrosis affecting both the compact and spongy ventricular and atrial muscle, with pyknotic nuclei, shrunken cells and hyaline degeneration. Infiltration and endocardial proliferation can also be observed [22,58,59]. However, the pathogenesis of the lesion has not been fully characterised, neither the involvement of the heart in the outcome of the disease been fully elucidated. The SCPCs model proposed here would be a valuable tool to gain insights in the mechanisms of cell death involvement in the development of these lesions.

Further immunological and histochemical characterisation of the SCPCs would be helpful to provide, for example, the proportion of myocytes to other cell types undoubtedly present in the culture, as well as their interactions and the degree of differentiation over the duration of cultivation [60].

In summary, the heart is a complex organ with unique characteristics and in order to study the mechanisms of infectious disease affecting it, the closer we can get to the species and tissues involved the more likely that the results will reflect events occurring in the whole animal. The *S*. *salar* SCPCs model proposed here represents a step forward in fish disease research by enabling the aforementioned complexity to allow host–pathogen interactions studies at the organ level under controlled conditions. Furthermore, SCPCs represent a bioethical and cost effective approach to improve our understanding of early events in the interaction between the host and a viral pathogen affecting the cardiac tissue. It can also be used to gather preliminary data to help design specific *in vivo* challenges, as well as providing platforms for testing potential chemotherapeutants. Successful cryopreservation of SCPCs, as well as further typing of the cell types and their evolution over time would present an obvious advantage and are a clear priority for further research.

## Supporting information

S1 Video Live SCPCs, ex vivo type. (MP4)

S2 Video Live SCPCs, de novo type. (MP4)

S1 QPCR data Source for graphics of the kinetics of infection. (XLSX)

S1 Fig TEM of cardiomyocyte intercalated disc. Direct magnification 8000x. Scale bar = 500nm.
(TIF)
