# Supplementary figures and images for: Atlantic salmon cardiac primary cultures: An in vitro model to study viral host pathogen interactions and pathogenesis
Source: PLoS One. 2017 Jul 20;12(7):e0181058. doi: 10.1371/journal.pone.0181058 (PMC5519056; doi:10.1371/journal.pone.0181058)

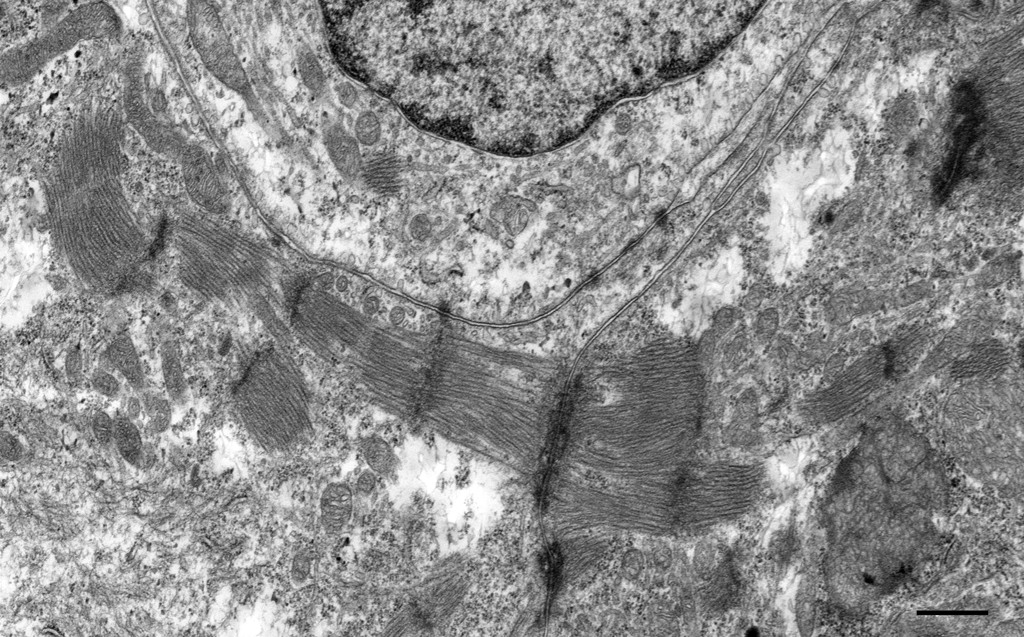

Supplement: S1 Fig — Direct magnification 8000x. Scale bar = 500nm. (TIF) [file pone.0181058.s004.tif]
